# Negation and Free Choice Inference in Child Mandarin

**DOI:** 10.3389/fpsyg.2020.591728

**Published:** 2020-10-26

**Authors:** Haiquan Huang, Peng Zhou, Stephen Crain

**Affiliations:** ^1^School of Foreign Languages, Hubei University of Technology, Wuhan, China; ^2^Department of Foreign Languages and Literatures, Tsinghua University, Beijing, China; ^3^Department of Linguistics, Macquarie University, Sydney, NSW, Australia

**Keywords:** free choice inference, child Mandarin, disjunction, internal negation, external negation

## Abstract

In sentences with internal negation, Free Choice Inferences (FCIs) are canceled (Chierchia, 2013). The present study investigated the possibility that FCIs are negated, not canceled, by external negation. In previous research, both Mandarin-speaking children and adults were found to license FCIs in affirmative sentences with a modal verb and the disjunction word *huozhe* ‘or’ (Zhou et al., 2013). The present study contrasted internal versus external negation in sentences that contained all the ingredients needed to license FCIs. Four experiments were conducted using the Truth Value Judgment Task (Crain and Thornton, 1998). Experiment 1 tested Mandarin-speaking children and adults using sentences with internal negation, a modal verb and disjunction. As expected, children did not license FCIs; rather, they assigned a ‘neither’ interpretation to disjunction. Also as expected, adults analyzed disjunction as taking scope over internal negation, yielding a ‘not both’ interpretation (Jing et al., 2005). Experiment 1 provided the benchmarks for sentences with external negation in Experiments 2-4. Experiment 2 confirmed that English-speaking adults distinguish between internal and external negation in sentences with disjunction. In Experiment 3, external negation was conveyed by the focus adverb *zhiyou* ‘only’. External negation eliminated the between-group differences observed in Experiment 1. Both children and adults analyzed external negation as taking scope over disjunction. Experiment 4 tested the effect of external negation on the computation of FCIs. The test sentences only differed from Experiment 1 by using external negation, rather than internal negation. Again, children and adults interpreted the test sentences in the same way. Most importantly, in contrast to Experiment 1 (with internal negation), both groups analyzed external negation as negating, rather than canceling, FCIs. The findings support the distinction between internal and external negation.

## Introduction

The present study investigates the empirical consequences of two kinds of negation. In the theoretical literature, these two negations are often referred to as predicate negation and propositional negation (see, e.g., Karttunen and Peters, 1979; Ladusaw, 1980; Bochvar and Bergmann, 1981; Horn, 1985, 2001; Schwarz and Bhatt, 2006; Bar-Asher Siegal, 2015). Adopting the terminology by Bar-Asher Siegal (2015), we will refer to them as internal and external negation. Internal and external negation have distinct syntactic distributions and make different semantic contributions to sentence meaning.

The present study extends the meaning differences attributed to the distinction between internal and external negation, in two ways. First, external negation cancels the polarity sensitivity of Positive Polarity Items (PPIs). Expressions that are analyzed by adult speakers as PPIs differ across languages. These cross-linguistic differences are revealed in sentences with internal negation, but not in sentences with external negation. That is, external negation cancels the polarity sensitivity of disjunction words. Second, external negation does not cancel Free Choice Inferences; rather it negates such inferences. This contrasts with internal negation, which cancels Free Choice Inferences (Chierchia, 2013). To document these contributions made to sentence meaning by external negation, we report the findings of four experiments. The first experiment demonstrates that the Mandarin Chinese word for disjunction, *huozhe* ‘or,’ is polarity sensitive for adults, but not for children. We show that adults analyze the disjunction word *huozhe* ‘or’ as taking scope over internal negation, whereas children interpret disjunction *in situ*, as in English. Like English-speaking children and adults, therefore, internal negation cancels Free Choice Inferences for Mandarin-speaking children. In summary, Experiment 1 documents two facts about internal negation: First, it interacts with polarity sensitive expressions and, second, it cancels Free Choice Inferences for speakers who interpret disjunction *in situ*.

The test sentences in Experiments 2-4 contain external negation. Experiment 2 establishes the expected meaning differences between internal and external negation in English, a language in which disjunction is not polarity sensitive (either for children or adults). Experiments 3 and 4 are the heart of the paper. These experiments investigate the interpretations that are assigned to sentences with external negation by Mandarin-speaking children and adults. We assess the effect of external negation on the polarity sensitivity of disjunction for adult speakers of Mandarin, and how external negation effects Free Choice Inferences by Mandarin-speaking children and adults. Our two main experimental hypotheses are the following:

1) External negation is predicted to cancel the polarity sensitivity of disjunction, such that Mandarin-speaking children and adults analyze disjunction in the same way.
2) External negation is expected to negate but not cancel Free Choice Inferences, both for Mandarin-speaking children and adults.

This concludes our introduction. The remainder of the paper is structured as follows. The second section introduces the preliminaries: internal versus external negation. The third section discusses Free Choice Inferences. The fourth section reviews the relevant acquisition data. The fifth section reports our experimental studies. The sixth section makes a general discussion and concludes the paper.

## Preliminaries: Internal Versus External Negation

Syntactically, internal negation typically occurs sentence-internally, in the predicate phrase, as in (1), whereas external negation typically appears in sentence-initial position, as in (2).

(1) It is true that John did not eat beef.
(2) It is not true that John ate beef.

One linguistic manifestation of the distinction between internal and external negation appears in sentences with Positive Polarity Items (PPIs). PPIs take scope over internal negation, but external negation takes scope over PPIs—PPIs are interpreted *in situ* in sentences with external negation (e.g., Baker, 1970; Ladusaw, 1980; Schwarz, 2004; Szabolcsi, 2004; Crain, 2012; Bar-Asher Siegal, 2015). For example, English *some* is a PPI. Therefore, *some* is interpreted as taking scope over negation in sentence (3), so the sentence can be paraphrased as: *There is some beef that John didn’t eat*. By contrast, *some* is interpreted *in situ* in sentences with external negation, as in (4). So, sentence (4) is truth conditionally equivalent to *John didn’t eat any beef*.

(3) It is true that John did not eat some beef.
(4) It is not true that John ate some beef.

To further clarify the distinction between internal and external negation, it will be instructive to discuss some further examples of negative sentences with internal versus external negation. These examples are sentences that contain so-called NEG-raising predicates such as *think* (*want, believe*), as compared to with other predicates such as *say* (*demand, claim*). NEG-raising predicates pose a potential problem in maintaining a clear distinction between internal and external negation. Consider sentences (5) and (6). In both sentences, negation is positioned in the matrix clause rather than in the embedded clause. So, both sentences appear to have external negation. Notice that an interpretation is available for sentence (5) that is not available for sentence (6).

(5) I don’t think that John eats beef.
(6) I didn’t say that John eats beef.

This interpretation can be paraphrased by repositioning negation from the matrix clause to the embedded clause, yielding an interpretation that can be paraphrased as “I think that John does not eat beef.” There is no corresponding interpretation for sentence (6). That is, there is no paraphrase of (6) that means “I said that John does not like beef.” The availability of this interpretation is why the predicate *think* is said to be NEG-raising. According to one analysis of NEG Raising, negation originated in the embedded clause of (5) and then moved to the matrix clause.^1^ Negation can be interpreted in either position, so (5) has two distinct interpretations, whereas (6) is unambiguous, with negation interpreted *in situ*.

This syntactic analysis of NEG Raising is supported by a difference in the acceptability of certain Negative Polarity Items in sentences with predicates like *think*, but not in sentences with predicate like *say*. In (7), for example, the NPI *ever* is licensed in the embedded clause, whereas *ever* is not licensed in (8). To explain this contrast, we can follow the NEG-raising analysis of (5). Negation originates in the embedded clause in (7), where it licenses *ever*, and subsequently moves to the matrix clause. On this analysis, the distinction between internal and external negation is blurred in sentence where predicates like *think* are negated.

(7) I don’t think that John has ever been to Melbourne.
(8) ^∗^I didn’t say that John has ever been to Melbourne.

There is no asymmetry in the interpretation of sentences with predicates like *think* and *say*, however, when the embedded clause contains a Positive Polarity Item (PPI). Negating either predicate cancels the polarity sensitivity of PPIs. To illustrate, consider the English PPIs “already” and “would rather.” Examples (9)-(12) show that both of these PPIs are unacceptable when they are clausemates with negation.

(9) ^∗^I think that John hasn’t already been to Melbourne.
(10) ^∗^I said that John hasn’t already been to Melbourne.
(11) ^∗^I think that John wouldn’t rather live in Victoria.
(12) ^∗^I said that John wouldn’t rather live in Victoria.

When negation is positioned in the matrix clause, however, these PPIs are acceptable in sentences with *think* and in sentences with *say*. This is illustrated in examples (13)-(16).

(13) I don’t think that John has already been to Melbourne.
(14) I didn’t say that John has already been to Melbourne.
(15) I don’t think that John would rather live in Victoria.
(16) I didn’t say that John would rather live in Victoria.

In summary, there is no blurring of the distinction between external and internal negation in sentences with PPIs. PPIs are licensed by external negation, but they are not licensed by internal negation.

Let us discuss one more example. Although the PPI “some” is acceptable in sentences with internal negation, it is interpreted as taking scope over negation. This inverse scope interpretation is characteristic of both NEG-raising predicates like “think” and non-NEG-raising predicates like “say.” This is illustrated in examples (17) and (18). In both examples, the truth conditions differ from the truth conditions that result if “some” is replaced by “any”.

(17) I think that John won’t eat some of the dessert.
Cf. I think that John won’t eat any of the dessert.
(18) I said that John won’t eat some of the dessert.
Cf. I said that John won’t eat any of the dessert.

By contrast, if negation appears in the matrix clause, as in examples (19) and (20), the truth conditions are not altered if “any” replaces “some.” This is because the PPI “some” is interpreted in the scope of external negation in both examples. Again, the polarity sensitivity of PPIs is canceled in sentences with external negation.

(19) I don’t think that John will eat some (any) of the dessert.
(20) I didn’t say that John will eat some (any) of the dessert.

In conclusion, NEG Raising might be seen as a threat to the distinction between internal and external negation. However, this distinction is not at issue in sentences with Positive Polarity Items. In languages where disjunction word is a PPI, then, we anticipate that disjunction phrases will be interpreted as taking scope over internal negation, but will be interpreted in situ in sentences with external negation, regardless of the predicate that appears in the matrix clause.

In one class of languages, the word for disjunction is not a Positive Polarity Item (PPI) such that it is interpreted *in situ* even if it occurs under internal negation. For example, the English disjunction word *or* is not a PPI, so it is interpreted *in situ* in sentences with internal negation. To illustrate, consider the English sentence in (21), where the disjunction word *or* appears under the (internal) negation marker *not*.

(21) John did not order sushi or pasta.

For both English-speaking adults and children, sentence (21) is judged to be true only in circumstances in which John did not order either sushi or pasta. This interpretation reveals that the English disjunction word *or* is not a PPI such that it is interpreted *in situ* at the level of semantics.

In another class of languages, the word for disjunction is a PPI (Goro and Akiba, 2004; Szabolcsi, 2004; Crain, 2012; Notley et al., 2012). For example, the Mandarin disjunction word *huozhe* ‘or’ is a PPI (Jing et al., 2005; Crain, 2012; Notley et al., 2012). Consider the Mandarin sentence in (22), where the (internal) negation marker is *mei* ‘not’.

(22) Yuehan mei^2^ chi shousi huozhe yidalimian.
John Neg eat sushi or pasta.

For adult speakers, sentence (22) is judged to be true in circumstances in which John only ate sushi, or John only ate pasta. This interpretation is a consequence of the fact that disjunction takes scope over negation in (22), so the sentence can be paraphrased in English using a cleft sentence: *It was pasta or sushi that John didn’t eat*.

In all languages, as far as we know, disjunction is interpreted *in situ* when it appears in sentences with external negation (see Baker, 1970; Ladusaw, 1980; Schwarz, 2004; Szabolcsi, 2004; Crain, 2012; Bar-Asher Siegal, 2015). For example, consider the Mandarin sentence (23), where the (external) negation marker is *bushi*^3^. Sentence (23) is judged to be true only in circumstances in which John did not eat either sushi or pasta. This interpretation reflects that fact that the polarity sensitivity of Mandarin disjunction is canceled by external negation such that it is interpreted *in situ*.

(23) Shishi bushi yuehan chi-le shousi huozhe yidalimian.
Fact Neg John eat-PERF sushi or pasta.
‘It’s not true that John ate sushi or pasta.’

Why is the polarity sensitivity of Mandarin disjunction canceled by external negation, not by internal negation? Based on the linguistic environments that cancel polarity sensitivity, there appear to be three prerequisites for an expression to be interpreted as a PPI (Szabolcsi, 2002; Crain and Khlentzos, 2008). First, the anti-licensing expression and the PPI must be clausemates. Second, the anti-licensor must c-command the PPI. Third, they must both be overt (phonologically realized). To illustrate, let’s reconsider the Mandarin sentence (22). In (22), the anti-licensor *mei* ‘not’ c-commands the disjunction word *huozhe* ‘or’ and both of the expressions appear in the same clause. In such a linguistic environment, the disjunction word *huozhe* is licensed as a PPI and thus interpreted as taking scope over internal negation. By contrast, when an anti-licensor occurs outside the clause that contains a PPI, as in sentences with external negation, the polarity sensitivity of the PPI is canceled by external negation. As a result, the PPI is interpreted *in situ*. To illustrate, let’s reconsider sentence (23). In (23), the anti-licensor *bu* ‘not’ appears outside the clause that contains the disjunction word *huozhe* ‘or’, so the positive polarity sensitivity of the disjunction word *huozhe* is not licensed by external negation. As a result, the disjunction word *huozhe* is interpreted *in situ*. In a nutshell, external negation cancels the polarity sensitivity of Mandarin disjunction, whereas internal negation doesn’t. One thesis of the present study is to see whether there is such a distinction between internal and external negation.

## Free Choice Inferences

The present study investigated another possible difference between internal and external negation, involving the licensing of Free Choice Inferences (Kamp, 1973, 1978).

A Free Choice Inference is licensed in sentence (24), where disjunction appears in the scope of the deontic modal verb *is allowed to*. Due to the presence of the modal verb, English-speaking adults judge (24) to be true if John is allowed to eat sushi and is allowed to eat pasta. The symbol ‘↝’ below (24) represents an inference, rather than an entailment.

(24) John is allowed to eat sushi or pasta.
↝ John is allowed to eat sushi and he is allowed to eat pasta.

Sentence (24) contains disjunction. It is surprising that sentence (24), with disjunction, licenses an inference that can be represented using conjunction. In standard logic, a formula with disjunction (Sj ∨ Pj) does not entail one with conjunction (Sj ∧ Pj), where ‘S’ represents sushi, ‘P’ represents pasta, and ‘j’ represents John. This is also true in human languages. If we remove the modal verb in (24), the sentence that results in is *John ate pasta or sushi*. In judging this statement, adult English speakers typically make an inference of exclusivity (viz, ‘not both’

interpretation); they judge the sentence to be true in circumstances in which John only ate sushi or only ate pasta. The inference of exclusivity is not generally regarded as being part of the basic meaning of disjunction but, rather, as being derived by an implicature (see e.g., Horn, 1972; Gazdar, 1979; Levinson, 1983, 2000; Chierchia et al., 2001; Sauerland, 2004). In modal logic, a disjunctive statement ♢ (Sj ∨ Pj), with the wide scope of the possibility operator ‘♢’, does not entail the corresponding formula with conjunction, ♢ Sj ∧♢ Pj. Clearly, the deontic modal verb *is allowed to* in (24) is responsible for licensing the FCI. For discussion, see von Wright (1969), Kamp (1973, 1978), Dayal (1998), Giannakidou (2001), Kratzer and Shimoyama (2002), Aloni and van Rooij (2004), Sauerland (2004), Geurts (2005), Schulz (2005), Alonso-Ovalle (2006), Chierchia (2006, 2013), van Rooij (2006, 2010), Aloni (2007), Fox (2007), Klinedinst (2007), Franke (2011).

It has been proposed that, like other inferences, Free Choice Inferences are canceled in sentences with internal negation (Chierchia, 2013, 2017). For example, sentence (25) entails that John is not allowed to eat sushi and that John is not allowed to eat pasta. So, sentence (25) generates a conjunctive entailment (the ‘neither’ interpretation), despite the presence of the modal expression *is allowed to*.

(25) John is not allowed to eat sushi or pasta.

Suppose a Free Choice Inference was generated in (25). Then, this inference would be negated. The result would be weaker than the ‘neither’ interpretation. A negated Free Choice Inference would result in a ‘not both’ interpretation: ∼ (♢Sj ∧♢Pj). The weaker interpretation would make sentence (25) true if John is only allowed to eat sushi, or if John is only allowed to eat pasta. Sentence (25) would also be true in the one circumstance that corresponds to a conjunctive entailment—that is, if John is not allowed to eat either dish. However, this is not an entailment, since it is just one of the truth conditions that would be available for sentence (25).

Free Choice Inferences are not canceled in sentences with external negation, however. To illustrate, consider the English sentence in (26).

(26) It is not true that John is allowed to eat sushi or pasta.

As the English disjunction word *or* is not a PPI, it is always interpreted *in situ* regardless of whether it appears in sentences with internal or external negation. Therefore, sentence (26) would be judged to be true if John is only allowed to eat sushi, or if he is only allowed to eat pasta. In contrast to sentence (25), the Free Choice Inference in sentence (26) is negated, rather than canceled.

We now turn to Mandarin Chinese. It has been found that disjunction is typically analyzed as a PPI in Mandarin (e.g., Jing et al., 2005; Crain, 2012; Notley et al., 2012). For adults, disjunction is interpreted as taking scope over internal negation at the level of semantics. By contrast, children initially do not analyze disjunction as a PPI. It has been reported, however, that Mandarin-speaking children initially interpret disjunction *in situ* when it appears in sentences with internal negation (e.g., Jing et al., 2005; Crain, 2012; Notley et al., 2012). Moreover, the polarity sensitivity of Mandarin disjunction is expected to be canceled by external negation. Therefore, both Mandarin speaking children and adults are expected to interpret disjunction *in situ* when it occurs in sentences with external negation. The different patterns of interpretations of disjunction by child and adult speakers of Mandarin play a critical role in generating a specific set of predictions about the inferences and entailments that Mandarin-speaking children and adults will generate in a wide range of linguistic structures. These predictions are pursued in a series of experiments. In particular, the series of experiments is designed to see how Mandarin-speaking children and adults interpret free choice permission constructions under internal and external negation.

## Child Language

This section reviews children’s computation of Free Choice Inferences in affirmative sentences, as well as children’s understanding of sentences with negation. We first review previous studies that have documented that young children compute FCIs in sentences that contain a modal verb and the disjunction word *huozhe* ‘or’ or sentences with the polarity sensitive item *renhe* ‘any’ (see e.g., Zhou et al., 2013; Huang and Crain, 2014; Tieu et al., 2016).

### Children’s Computation of Free Choice Inferences

In previous studies, preschool Mandarin-speaking children were found to compute Free Choice Inferences in affirmative sentences (Zhou et al., 2013; Huang and Crain, 2014; Tieu et al., 2016). A representative study is by Zhou et al. (2013). Using a Truth Value Judgment Task (Crain and Thornton, 1998), these researchers investigated Mandarin-speaking children’s interpretation of affirmative sentences that contained the disjunction word *huozhe* ‘or’ and the deontic modal verb *keyi ‘may*.’ On a typical trial, Kung Fu Panda and Batman had a car-pushing competition. Mr. Owl was the judge of the competition, so he proclaimed that Kung Fu Panda was permitted to push the green car, but not the orange car and that Batman was permitted to push the orange car, but not the green car. Being absent-minded, the puppet didn’t hear the proclamations clearly, so he described the story using the test sentence with disjunction, as in (27).

(27) Gongfu xiongmao keyi tui lüse de xiaoche huozhe juse de xiaoche.
Kung Fu Panda may push green car or orange car
‘Kung Fu Panda may push the green car or the orange car.’

(28) ↝ Kung Fu Panda may push the green car and he may push the orange car.

The child participants rejected sentences like (27) in the similar contexts 95% of the time. On the typical trial, for example, the child participants justified their rejections by making reference to the fact that Kung Fu Panda was only allowed to push one of the cars. This revealed that the child participants computed FCIs, as indicated in (28), from sentences like (27).

Using the same methodology, Huang and Crain (2014) also found that Mandarin-speaking children computed (universal) Free Choice Inferences in affirmative sentences that contained the polarity sensitive expression *renhe* ‘any’ and the abilitative modal verb *neng* ‘is able to’. On a typical trial, Kung Fu Panda and Grasshopper participated in a car-pushing competition and a fence-jumping competition. In each competition, the two characters had the opportunity to try three different objects. In the car-pushing competition, Grasshopper successfully pushed one small car, but he failed with the other two big ones. At that point, the puppet produced test sentence (29). The child’s task was to judge whether or not the puppet had said the right thing. Sentence (29) did not contain *renhe* ‘any’, and it was a true description of the story, so children were expected to accept it.

(29) Zhameng neng tuidong yi-ge chezi.
Grasshopper can push one-CL car
‘Grasshopper was able to push one of the cars.’

Following the child’s assessment of the puppet’s statement (29), the story continued. Kung Fu Panda successfully pushed two cars, but he failed with the biggest one. Then, the puppet produced test sentence (30).

(30) Gongfuxiongmao neng tuidong renhe yi-ge chezi.
Kung Fu Panda can push any one-CL car
‘Kung Fu Panda was able to push any one of the cars.’

In contrast to (29), (30) contained *renhe* ‘any’, which resided in the scope of the abilitative modal verb *neng*. This configuration gives rise to a (universal) Free Choice Inference. That is, Kung Fu Panda was able to push all of the cars on offer. If children computed the FCI, they were expected to reject (30) since Kung Fu Panda failed to push the biggest car.

The story continued. Because Grasshopper failed in the first competition, he proposed to have a fence-jumping competition. In the second competition, Kung Fu Panda successfully jumped over a small fence, but failed to jump over the other two big ones. Then, the puppet produced test sentence (31).

(31) Gongfuxiongmao neng tiaoguo renhe yi-ge zhalan.
Kung Fu Panda can jump any one-CL fence
‘Kung Fu Panda was able to jump over any one of the fences.’

Like (30), (31) gives rise to a Free Choice Inference. That is, Kung Fu Panda was able to jump over all of the fences. If children computed the FCI, they were expected to reject (31) as Kung Fu Panda failed to jump over two of the fences. Finally, Grasshopper jumped over all of the three fences. Then, the puppet produced test sentence (32).

(32) Zhameng neng tiaoguo renhe yi-ge zhalan.
Grasshopper can jump any one-CL fence
‘Grasshopper was able to jump over any one of the fences.’

Sentence (32) gives rise to a Free Choice Inference. If children computed the FCI, they were expected to accept (32) since Grasshopper successfully jumped over all of the fences.

The findings were exactly as anticipated. Children accepted test sentences like (29) 100% of the time, but rejected ones like (30) 82% of the time. This pattern of responses revealed that children understood the semantic contribution of *renhe* ‘any’, as test sentences like (29) and (30) were presented in the similar scenarios and they differed only in the presence or absence of *renhe* ‘any’. In addition, children rejected test sentences like (31) 83% of the time, but accepted ones like (32) 83% of the time. The main finding was that Mandarin-speaking children compute (universal) FCIs from affirmative sentences that contain the polarity sensitive expression *renhe* ‘any’ embedded under the abilitative modal verb *neng* ‘is able to’.

The two studies reviewed in this section invite the conclusion that preschool children are able to compute Free Choice Inferences in affirmative sentences. Compared to previous research that used affirmative sentences, the present study attempts to investigate Mandarin-speaking children’s computation of FCIs in sentences with negation. So, the next section reviews the previous studies on children’s understanding of sentences with negation.

### Children’s Understanding of Sentences With Negation

Previous research has reported that negative sentences are more difficult to process than their affirmative counterparts (see e.g., Wason, 1959; Sherman, 1976; Carpenter et al., 1999). Nevertheless, several other studies have shown that both children and adults have no difficulty understanding negative sentences when they are presented in felicitous contexts (see e.g., de Villiers and Tager-Flusberg, 1975; Crain et al., 1996; Gualmini, 2004; Staab, 2007). A representative study is by de Villiers and Tager-Flusberg (1975). Using a sentence completion task, these researchers investigated 2- to 5-year old English-speaking children’s understanding of negative sentences. On a typical trial, the child was presented with some objects (or drawings), among which one differed from the other six or seven items (e.g., one baby’s bottle and seven cars). After that, the experimenter pointed at one of the objects and asked the child: “This is a __?” or “That is not a ___?”. These two questions were designed to elicit a true affirmative or a true negative. In such contexts, it is more plausible to answer a negative probe for a different item (e.g., the baby’s bottle) than for a similar item (e.g., one of the seven cars). This hypothesis was confirmed by the experimental results. The child participants only had difficulty in responding to implausible negative statements. The findings suggest that negation does not pose any processing challenges for young children once it is used in felicitous contexts. This study lends further support for the argument that negation per se does not cause any processing difficulties (Horn, 1989).

In addition, several cross-linguistic studies have shown that preschool children understand sentences with covert external negation, introduced by a focus adverb, i.e., *only* (Goro et al., 2005; Minai et al., 2006; Zhou and Crain, 2010). To understand the experimental design, let’s first consider the semantics of sentences with the focus adverb *only*, as illustrated in (33).

(33) Only Bunny Rabbit ate a carrot or a pepper.
**Presupposition:** Bunny Rabbit ate a carrot or a pepper.
**Assertion:** It is not true that anyone other than Bunny Rabbit ate a carrot or a pepper.

The focus adverb *only* is typically associated with some expression in a sentence, called the focus element, which more often than not receives phonological stress. In (33), the focus element is *Bunny Rabbit*. In addition to its association with the focus element, the focus adverb *only* contributes two meaning components: one is positive, and the other is negative (see e.g., Horn, 1969; Anderson, 1972; Jacobs, 1983; Rooth, 1985, 1992; van Stechow, 1990; Beaver et al., 2017). The positive meaning component is called the presupposition, which expresses the content of the original sentence, without the focus adverb *only*. That is, *Bunny Rabbit ate a carrot or a pepper*. The negative meaning component is called the assertion, which pertains to a set of individuals (or predicates) being contrasted with the focus element. The assertion entails it’s not the case that anyone other than Bunny Rabbit ate a carrot or a pepper. In this sense, the focus adverb *only* introduces covert external negation.

Using a Truth Value Judgment Task, Goro et al. (2005) assessed English-speaking children’s understanding of the presupposition and assertion meaning components of sentences like (33). For our purposes, the relevant findings are how children interpreted the assertion meaning component of sentences like (33). In one condition, the child participants were presented with sentence (33) in a situation where Bunny Rabbit only ate a carrot, Winnie the Pooh ate nothing and Cookie Monster only ate a pepper. The assertion entails it’s not true that anyone other than Bunny Rabbit ate either a carrot or a pepper. The result is a ‘neither’ interpretation, which is inconsistent with the fact that Cookie Monster ate a pepper. Therefore, the child participants were expected to reject sentence (33) if they accessed the assertion meaning. As anticipated, the child participants rejected sentences like (33) 90% of the time in the similar contexts. Minai et al. (2006) and Zhou and Crain (2010), respectively, investigated the linguistic structure with Mandarin-speaking children and Japanese-speaking children, and similar patterns of responses were observed across the two languages. The findings invite the conclusion that children understand sentences with covert external negation, introduced by a focus adverb.

The studies reviewed in this section invite us to reach three conclusions. First, children are able to compute Free Choice Inferences in affirmative sentences. Second, children have no difficulty in understanding negative sentences if they are presented in felicitous contexts. Third, children are able to comprehend sentences with covert external negation, introduced by a focus adverb, i.e., *only*. Besides these acquisition profiles, it has been proposed in the theoretical literature, that Free Choice Inferences are canceled in sentences with (internal) negation (Chierchia, 2013). Against this background, the present study was designed to contrast internal versus external negation in sentences that included all the ingredients needed to license FCIs. The main thesis was to see whether Mandarin-speaking children and adults cancel FCIs in sentences with internal negation, but negate rather than cancel such inferences in sentences with external negation.

## Experiments

### Experiment 1

Experiment 1 assessed the interpretation that Mandarin-speaking children and adults assigned to sentences with internal negation, a modal verb and disjunction. Before introducing the typical test sentence, let’s consider a simple English sentence with internal negation and disjunction, as in (34).

(34) Jack did not eat pasta or sushi.

Sentence (34) generates a ‘neither’ interpretation. A straightforward translation of sentence (34) in Mandarin is (35).

(35) Jieke mei chi yidalimian huozhe shousi.
Jack Neg eat pasta or sushi

For adult speakers of Mandarin, sentence (35) expresses the interpretation conveyed by the following English sentence: *Jack didn’t eat pasta or Jack didn’t eat sushi.* That is, adult speakers of Mandarin analyze the disjunction word *huozhe* ‘or’ as a Positive Polarity Item and interpret it as taking scope over internal negation (see e.g., Jing et al., 2005; Crain, 2012; Notley et al., 2012). This results in a ‘not both’ interpretation. In contrast to adults, children initially do not analyze the disjunction word *huozhe* ‘or’ as a PPI (see e.g., Jing et al., 2005; Crain, 2012; Notley et al., 2012). For children, negation takes scope over disjunction in sentences like (35). The result is a ‘neither’ interpretation, as in English.

Experiment 1 was designed to test the experimental hypothesis, based on previous research, that Mandarin-speaking children, but not Mandarin-speaking adults, will cancel FCIs in sentences with internal negation. A typical test sentence is illustrated in (36), which contained the disjunction word *huozhe* ‘or’, the deontic modal verb *beiyunxu* ‘is allowed to’ and the negation marker *mei* ‘not’.

(36) Zhangsan mei beiyunxu chi yidalimian huozhe jirou.
Zhangsan Neg PM allow eat pasta or chicken

The experimental hypothesis is that Mandarin-speaking children will interpret sentence (36) in the same way English-speaking children and adults do. That is, children are expected to cancel the Free Choice Inference, and interpret (36) to mean that Zhangsan was not allowed to eat either pasta or chicken. However, the fact that the Mandarin disjunction word *huozhe* ‘or’ is a PPI for adults leads to a different hypothesis. If the disjunction word *huozhe* ‘or’ is analyzed as residing outside the scope of negation, then the Free Choice Inference will not be canceled. On this scenario, Mandarin-speaking adults will take (36) to mean that either Zhangsan was not allowed to eat pasta, or he was not allowed to eat chicken. On this interpretation, Mandarin-speaking adults are expected to accept (36) in circumstances in which Zhangsan was only allowed to eat pasta, or he was only allowed to eat chicken.

#### Participants

Twenty-two Mandarin-speaking children participated in the experiment, and they ranged in age from 4;9 (years; months) to 5;8, with an average age of 5;4. The child participants were recruited from a kindergarten affiliated with the Hubei University of Technology (HBUT), Wuhan, China. We also tested twenty Mandarin-speaking adults, who were undergraduates at HBUT.

#### Procedures

Participants were presented with a Truth Value Judgment Task. Two experimenters were involved in the task. One experimenter acted out stories with toy characters and props, while the other experimenter manipulated a puppet, Kermit the frog. The child watched the acted-out stories alongside Kermit. At the end of each story, Kermit described what had happened in the story, using a test sentence. The child’s task was to judge whether Kermit’s description was right or wrong. If the child indicated what Kermit had said was wrong, then s/he was asked to explain what had really happened in the story.

The child participants were first introduced to the task as a group, and then they were tested individually in a quiet room. Before the main test session, a practice trial was designed to familiarize the child with the task. As the target sentences contained the infrequent deontic modal verb *beiyunxu* ‘is allowed to’, the practice trial was also designed to see whether the child understood the semantic meaning of the deontic modal verb. On the practice trial, there was a minimal pair of sentences, one with the deontic modal verb *beiyunxu* ‘is allowed to’, as illustrated in (37), and the other without the deontic modal verb *beiynxu*, as indicated in (38). The presence or absence of the deontic modal verb *beiyunxu* ‘is allowed to’ leads to different truth-values in Mandarin. The correct interpretations of the two sentences were then taken as indicators that the child understood the task as well as the semantic meaning of the deontic modal verb *beiyunxu*.

(37) Xiaogou beiyunxu chi jirou le.
Small dog PM allow eat chicken le
‘The small dog was allowed to eat chicken.’

(38) Xiaogou chi jirou le.
Small dog eat chicken le
‘The small dog ate chicken.’

The child was present with the two sentences in scenarios in which the small dog turned out to eat chicken even though he was only permitted to eat fish, but not chicken. It was expected that the child would reject (37), but accept (38). All of the child participants responded correctly on the practice trial. Moreover, they justified their rejections of (37) on the grounds that the small dog was permitted to fish, but not chicken. The findings indicated the child participants clearly understood the task as well as the semantic meaning of the deontic modal verb *beiyunxu* ‘is allowed to’. Therefore, they were allowed to proceed to the main test session that contained four trials. On each trial, the child participants’ responses were recorded for subsequent data analysis.

The adult participants were directly tested on the same four trials as children, using a videotaped version. They were tested in groups of five at a time and were asked to respond individually on an answer sheet. On each trial, the adult participant was asked to judge whether Kermit had said ‘the right thing’. If s/he judged that Kermit was wrong, s/he was asked to provide his/her justifications.

#### Materials

Participants heard a total of eight sentences: Four were targets such as (36) and four were clearly true fillers such as (39).

(39) Wo zhidao yijian shiqing: Zhangsan beiyunxu chi shousi.
I know one-CL thing: Zhangsan PM allow eat sushi
‘I know one thing: Zhangsan was allowed to eat sushi.’

The eight sentences were evenly distributed across four test stories so that after each story, participants judged one target and one true filler. The presentation of target and filler items were in a pseudo-random order. To illustrate, here is a typical story.

**FIGURE 1:**
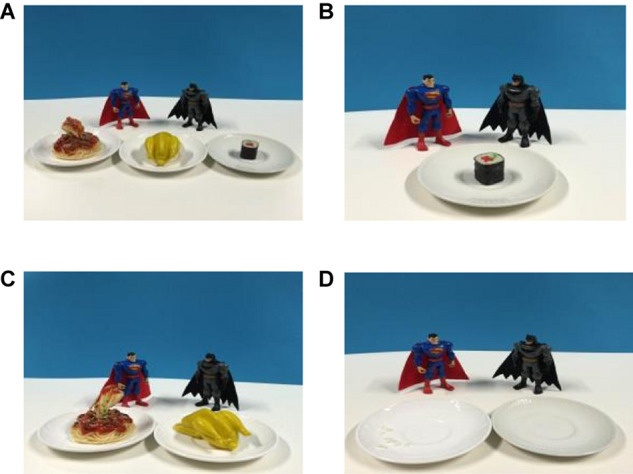
**(A)** Food Options, **(B)** Rule One,**(C)** Rule Two, **(D)** The Last Scene.

This is a story about Batman and Superman. Batman was training to become a better superhero, and he had asked Superman to help him get in shape. Superman said, “Batman, if you want to be a better superhero, you have to lose weight. You are eating too much, and you must go on a strict diet. For today’s lunch, there are three dishes: sushi, pasta and chicken (see Figure 1-A). Batman, you are only allowed to eat sushi. You cannot eat pasta, and you cannot eat chicken” (see Figure 1-B). But Batman said, “Superman, I can’t just eat sushi. I will be too weak to be a superhero. Please let me eat one more thing.” Superman said, “OK, Batman, you are allowed to eat one more thing: there is pasta and there is chicken. You can choose one of them, but not both” (see Figure 1-C). Batman was a shy boy, so he took the three dishes into a dining room, where he could hide up his table manners. Batman said to himself: “I love sushi very much, so I will eat the sushi first. Hmmm, the sushi is yummy!” After that, he ate a second dish and returned with two empty plates to show Superman (see Figure 1-D). Batman said, “Superman, I am still hungry. Can I eat the third dish?” Superman replied, “No, you will gain weight if you eat too much.” Batman said reluctantly, “OK, Superman, I will follow your instructions!”

When the story concluded, the experimenter asked Kermit to say what Batman was allowed to eat. At that point, Kermit produced the filler sentence (40).

(40) Wo zhidao yijian shiqing: Bianfuxia beiyunxu chi shousi.
I know one-CL thing: Batman PM allow eat sushi
‘I know one thing: Batman was allowed to eat sushi.’

Following that, the experimenter asked Kermit to say what Batman wasn’t allowed to eat at the end of the story. Then, Kermit presented the target sentence (41).

(41) Bianfuxia mei beiyunxu chi yidalimian huozhe jirou.
Batman Neg PM allow eat pasta or chicken
(a). **Expected child interpretation:** ‘Batman wasn’t allowed to eat pasta or chicken.’
(b). **Expected adult interpretation:** ‘Batman was not allowed to eat pasta, or he was not allowed to eat chicken.’

Since Batman had eaten the second dish in the dining room, Kermit and the participant didn’t witness what exactly he ate. Therefore, it was felicitous for Kermit to utter the target sentence with the disjunction word *huozhe* ‘or’ as he was ignorant of which dish (pasta or chicken) Batman didn’t eat.

The target sentences were expected to receive negative judgments, so it was important for us to ensure that our test stories met the felicity conditions for the use of negation (cf. Crain and Thornton, 1998). For this purpose, we adopted the research strategy advocated by Crain et al. (1996), who refer to the felicity conditions associated with negation as the Condition of Plausible Dissent. According to the Condition of Plausible Dissent, a negative judgment is appropriate only when the corresponding positive judgment is under consideration in the discourse context. To satisfy this condition, all of the test stories included a discrepancy that was created between the possible outcome and the actual outcome. For example, in the given story, it was made clear to the participants that it was possible that Batman wouldn’t be allowed to eat pasta or chicken. The actual outcome was that Batman was allowed to eat either pasta or chicken. This manipulation enabled us to satisfy the Condition of Plausible Dissent.

#### Predictions

The child participants were expected to compute a conjunctive entailment (the ‘neither’ interpretation) if they canceled Free Choice Inferences of the target sentences. For example, children were expected to interpret (41) as having the meaning in (41a). Therefore, they were expected to reject (41), because the meaning in (41a) is inconsistent with the actual outcome—Batman was allowed to eat either pasta or chicken. Adults were expected to assign a ‘not both’ interpretation, because disjunction is analyzed as a PPI in adult Mandarin. So, the adult participants were expected to interpret (41) as having the meaning in (41b). Therefore, they were expected to accept (41), because the meaning in (41b) is consistent with the actual outcome. The filler sentences such as (40) were clearly true, so both children and adults were expected to accept them. Considered together, the expected ‘Yes’ and ‘No’ responses from children were counterbalanced across trials.

#### Results and Discussion

**FIGURE 2:**
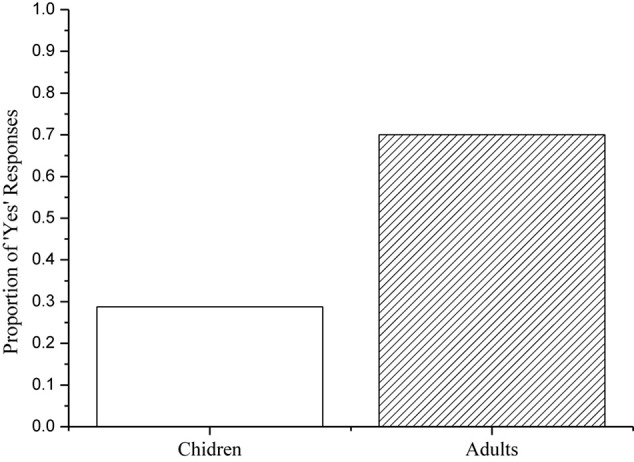
Children’s and adults’ percentages of ‘Yes’ responses to the target sentences.

Two child participants were excluded for failing to score 80% correct on the true fillers, leaving a total of 20 child participants.^4^ All of the adult participants responded correctly to the fillers 100% of the time, so the data from 20 children and 20 adults were included in the final analysis. A summary of their responses^5^ to the target sentences is provided in Figure 2.

As Figure 2 indicates, the child participants rejected the target sentences 71% of the time. Moreover, they provided plausible justifications for their rejections. For example, the child participants rejected (41) on the grounds that Batman was allowed to eat either pasta or chicken. This is compelling evidence that the child participants interpreted disjunction *in situ* in sentences with internal negation, resulting in a ‘neither’ interpretation. By contrast, the adult participants accepted the target sentences 70% of the time. This reveals that the adult participants interpreted disjunction as taking scope over internal negation, resulting in a ‘not both’ interpretation. A Mann-Whitney test on the No-responses to the target sentences revealed a significant difference between groups (*z* = 2.499, *p* = 0.012). The child participants computed a conjunctive entailment (the ‘neither’ interpretation) in response to the target sentences 71% of the time. This finding suggests that the child participants cancel FCIs in sentences with internal negation. By contrast, the adult participants assigned a ‘not both’ interpretation to the target sentences 70% of the time. This reflects the fact that disjunction is analyzed as a PPI by the adults. Nevertheless, 4 of the 20 children consistently assigned a ‘not both’ interpretation to the target sentences, suggesting that they have already converged on adult-like interpretation. Another 3 of the 20 children assigned a ‘not both’ interpretation 25% of the time, indicating that they have a slight tendency to converge on adult-like interpretation. In addition, 4 of the 20 adults consistently computed a conjunctive entailment, suggesting that they do not analyze Mandarin disjunction as a PPI. Another adult participant assigned a ‘neither’ interpretation 50% of the time, suggesting that he has a scope ambiguity between disjunction and internal negation. To some extent, the findings corroborated the cross-linguistic studies on both children’s and adults’ interpretations of simple negative sentences with disjunction (see e.g., Goro and Akiba, 2004; Crain, 2012; Notley et al., 2012).

In summary, Experiment 1 revealed two facts about internal negation. First, it interacts with the polarity sensitive expression *huozhe* ‘or’. Second, internal negation cancels FCIs for children who initially interpret the disjunction word *huozhe* ‘or’ *in situ*. These findings provided the benchmarks for sentences with external negation in Experiments 2-4.

### Experiment 2

Experiment 2 examined the meaning differences between internal and external negation in English, a language in which disjunction is not a PPI for either children or adults. As discussed earlier, internal negation and external negation make distinct contributions to sentence meaning. To illustrate, consider the English examples in (42) and (43).

(42) It is true that John is not allowed to eat sushi or pasta.
(43) It is not true that John is allowed to eat sushi or pasta.

Sentence (42) contains internal negation and disjunction, whereas sentence (43) contains external negation and disjunction. In English, the disjunction word *or* is not a PPI, so it is interpreted *in situ* regardless of whether it appears in sentences with internal or external negation. Therefore, sentence (42) is true in only one circumstance in which John is not allowed to eat either sushi or pasta. By contrast, sentence (43) is true in three circumstances: where John is only allowed to sushi; where John is only allowed to eat pasta; and where John is not allowed to eat either sushi or pasta. Sentence (42) illustrates that Free Choice Inferences are canceled in sentences with internal negation. Sentence (43) illustrates that FCIs are negated but not canceled in sentences with external negation, though sentences with external negation are true in circumstances that correspond to those in which FCIs are canceled.

So far, no empirical studies have been conducted to investigate the different semantic contributions by internal versus external negation. Therefore, Experiment 2 was designed to contrast English-speaking adults’ interpretation of sentences with internal versus external negation, as illustrated in (42) and (43).

#### Participants

Forty-two adult native-speakers of English were recruited through Amazon Mechanical Turk, and were paid $1 for their participation in the 10-minute experiment.

#### Procedure

The experiment was implemented and hosted on the Qualtrics platform. Participants were presented with an ‘adult’ version of the Truth Value Judgment Task. In the task, the participant was asked to read a series of short stories. After each story, s/he read the puppet’s two descriptions of the story (each description was one sentence long). The participant’s task was to judge whether or not the puppet said ‘the right thing’ about the story. If the participant judged that the puppet was right, then s/he was asked to click the ‘Yes’ button. Alternatively, if the participant judged that the puppet was wrong, s/he was asked to click the ‘No’ button.

#### Materials

There was a total of 16 sentences. Four sentences were internal negation targets like (42), four were external negation targets like (43), and there were eight fillers. Four of the fillers were clearly true, as in (44) and four were clearly false, as in (45).

(44) It is true that Jack is allowed to eat a cracker.
(45) It is true that Jack is only allowed to eat pasta.

The 16 sentences were evenly arranged into four different stories such that each story contained one internal negation target, or one external negation target, as well as one true filler or one false filler. To avoid carry-over effects, we adopted a between-subject design. 21 participants saw the internal negation targets. After each story, this group judged one test sentence with internal negation and one true filler. We will refer to these 21 participants as the internal negation group. The remaining 21 participants judged one test sentence with external negation and one false filler. These participants will be called the external negation group. Target and filler items were presented in a pseudo-random order. To illustrate, here is a typical trial.

Mr. Tiger, Mr. Horse, and Mr. Hippo are weight-lifting athletes, and their diet is strictly monitored by their coach. It is lunch time. The coach explains to each of the athletes what he is allowed to eat, and what he is not allowed to eat. The coach says: “for today’s lunch, there is sushi and pasta. There is also a cracker for a snack. I know everyone loves sushi and pasta. However, I will tell you what you can eat and what you cannot eat. OK, Mr. Tiger, let me look at you. It seems that you are gaining weight. You could eat nothing. However, you have tried very hard to control your weight. I don’t want you to become weak, so you can eat sushi. You cannot eat pasta because it may increase your weight. (The coach puts the sushi in front of Mr. Tiger.) Mr. Horse, let me look at you next. Mr. Horse, you are looking very fit. You are doing a good job with your training. So, you can eat sushi. You can eat pasta. It’s up to you. (The coach puts the sushi and the pasta in front of Mr. Horse.) Mr. Hippo, you are next. Mr. Hippo, you are exceeding your weight class. You are eating too much. So, you cannot eat sushi. You cannot eat pasta. You can have a cracker”. (The coach puts the cracker in front of Mr. Hippo.)

Mr. Tiger Mr. Horse Mr. Hippo
s s and p c
Kermit says: “I know what happened in the story…”

At that point, the internal negation group judged the target in (46) and the true filler in (47).

(46) It is true that Mr. Tiger is not allowed to eat sushi or pasta.
(47) It is true that Mr. Hippo is allowed to eat a cracker.

By contrast, the external negation group judged the target in (48) and the false filler in (49).

(48) It is not true that Mr. Tiger is allowed to eat sushi or pasta.
(49) It is true that Mr. Horse is only allowed to eat pasta.

As the target sentences also involved negative judgments, it was important for us to ensure that our test stories met the Condition of Plausible Dissent. As in Experiment 1, all of the test stories contained a discrepancy that was created between the possible outcome and the actual outcome. In the example story, the possible outcome was that Mr. Tiger would not be allowed to eat sushi and he would not be allowed to eat pasta. The actual outcome was that Mr. Tiger was allowed to eat sushi. Adding the possible outcome enabled us to satisfy the Condition of Plausible Dissent.

#### Predictions

The internal negation group were expected to compute a conjunctive entailment (the ‘neither’ interpretation) if they canceled Free Choice Inferences in sentences with internal negation. For example, they were expected to assign a ‘neither’ interpretation to (46) if they canceled the FCI. Therefore, they were expected to reject (46) because the ‘neither’ interpretation was inconsistent with the fact that Mr. Tiger was permitted to eat sushi. By contrast, the external negation group were expected to generate a ‘not both’ interpretation if they negated FCIs in sentences with external negation. For example, they were expected to assign a ‘not both’ interpretation to (48) if they negated the FCI^6^. Therefore, they were expected to accept (48) as the ‘not both’ interpretation was consistent with the fact that Mr. Tiger was permitted to eat sushi. The internal group was expected to accept the true fillers such as (47), but the external group was expected to reject the false fillers such as (49). Taken together, the expected ‘Yes’ and ‘No’ responses were counterbalanced across trials.

#### Results and Discussion

**FIGURE 3:**
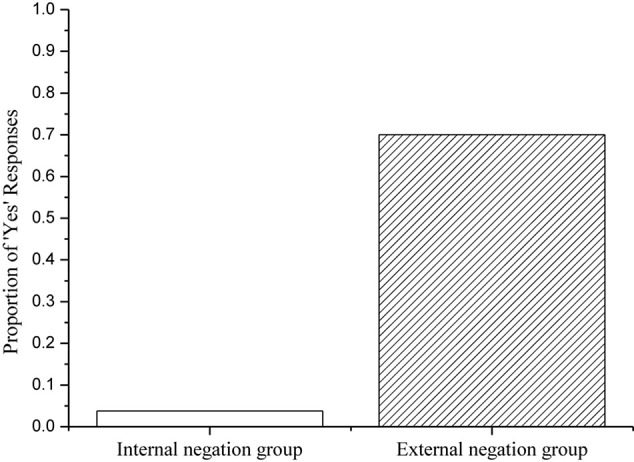
English-speaking adults’ percentages of ‘Yes’ responses to the target sentences.

Two participants were excluded for failing to score 80% correct on the true/false fillers, leaving 20 participants in the internal negation group and 20 in the external negation group. A summary of the responses by both groups to the target sentences is provided in Figure 3.

As Figure 3 shows, the internal negation group rejected the target sentences 96% of the time, whereas the external negation group accepted the target sentences 70% of the time. A Mann Whitney test on the No-responses to the target sentences revealed a significant effect between groups (*Z* = 4.278, *p* < 0.001). We can explain the findings as follows. When they interpreted sentences with internal negation, English-speaking adults computed a conjunctive entailment (the ‘neither’ interpretation). When they interpreted sentences with external negation, by contrast, English-speaking adults negated the Free Choice Inferences, yielding the ‘not both’ interpretation. It is worth noting, however, that English-speaking adults appear to have canceled the FCIs 30% of the time in sentences with external negation. Presumably, the English-speaking adults who gave this response computed a conjunctive entailment regardless of the position of negation in the target sentences. In summary, the findings confirmed the expected semantic differences between internal and external negation in English.

We extended this line of research to Mandarin Chinese in another two experiments. Specifically, we were interested to see if, like English-speaking adults, Mandarin-speaking children negate, but not cancel Free Choice Inferences in sentences with external negation. For this purpose, Experiments 3 and 4 were designed to contrast minimal pairs of sentences, namely disjunctive sentences with *zhiyou* ‘only’^7^ alone versus ones with *zhiyou* ‘only’ and the deontic modal verb *keyi* ‘is allowed to’. A typical minimal pair of test sentences is illustrated in (50) and (51).

(50) Zhiyou^8^ Zhangsan chi-le yidalimian huozhe shousi.
Only Zhangsan eat-ASP pasta or sushi
‘Only Zhangsan ate pasta or sushi.’

(51) Zhiyou Zhangsan keyi chi yidalimian huozhe shousi.
Only Zhangsan may eat pasta or sushi
‘Only Zhangsan is allowed to eat pasta or sushi.’

In contrast to English, the disjunction word *huozhe* ‘or’ is a PPI in Mandarin Chinese. It has been proposed that external negation cancels polarity sensitivity of linguistic expressions (Szabolcsi, 2002, 2004). If the polarity sensitivity of the disjunction word *huozhe* ‘or’ was canceled by external negation, both Mandarin-speaking children and adults would be expected to assign a conjunctive entailment (the ‘neither’ interpretation) to sentences like (50). This experimental hypothesis was evaluated in Experiment 3.

### Experiment 3

Experiment 3 was designed to investigate children’s interpretation of zhiyou only as introducing external negation to sentences with disjunction alone, which served a preliminary step to probe children’s computation of FCIs in sentences that contained an extra element, namely a modal verb, in Experiment 4.

#### Participants

Twenty-six Mandarin-speaking children were interviewed, and they ranged in age from 4;2 to 5;2, with a mean age of 4;7. The child participants were recruited from a kindergarten affiliated with Beijing Language and Culture University, Beijing, China. We also tested 20 Mandarin-speaking adults, who were undergraduates at Hubei University of Technology, Wuhan, China.

#### Procedures

Participants were tested using the same methodology and procedures as Experiment 1.

#### Materials

Participants heard a total of eight sentences: Four were targets, and four were fillers. The eight sentences were evenly distributed across four test stories so that after each story, participants judged one target and one filler, which were presented in a pseudo-random order. To illustrate, here is a typical story.

**FIGURE 4:**
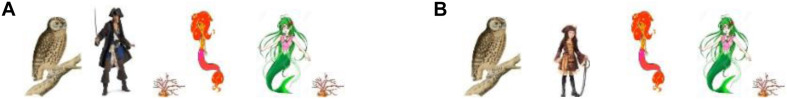
**(A)** The planting rules for big pirate. **(B)** The planting rules for small pirate.

*This is a story about a big pirate and a small pirate. The big pirate and the small pirate had a coral-planting game. Mr. Owl was the judge. He set the rules first. Mr. Owl said to the big pirate “Big Pirate, you are very strong, so you are allowed to plant corals near the red mermaid and you are allowed to plant corals near the green mermaid”* (see Figure 4-A). *Mr. Owl then said to the small pirate “Small Pirate, you are much weaker than Big Pirate, so you are allowed to plant corals near the green mermaid, but you are not allowed to plant corals near the red mermaid”* (see Figure 4-B). *Both the big pirate and the small pirate were very forgetful. They forgot about the rules when they were about to start the game. Therefore, they asked the puppet to remind them of the rules. After that, the story resumed. The big pirate planted corals near the red mermaid and the green mermaid, and the small pirate planted corals near the green mermaid.*

When the story concluded, the puppet said: “I wasn’t paying attention just now, so I don’t remember what exactly happened in the story. But I guess…”^9^ At that point, he produced the target (52) and the filler sentence (53).

(52) Zhiyou dahaidao zai hongse huozhe lüse meirenyu bianshang zhong-le shanhu.
Only big pirate at red or green mermaid side plant-ASP coral
‘Only the big pirate planted corals near the red mermaid or the green mermaid.

(53) Zhiyou xiaohaidao zai lüse meirenyu bianshang zhong-le shanhu.
Only small pirate at green mermaid side plant-ASP corals
‘Only the small pirate planted corals near the green mermaid.’

#### Predictions

We anticipated that participants would generate a conjunctive entailment (the ‘neither’ interpretation) in response to target sentences such as (52). As discussed earlier, the focus adverb *zhiyou* ‘only’ in (53) contributes two meaning components: A presupposition and an assertion. The presupposition expresses that the big pirate planted corals near the red mermaid or the green mermaid. The assertion entails that it’s not true that anyone other than the big pirate planted corals near the red mermaid or the green mermaid. In this sense, the focus adverb introduces covert external negation. If external negation cancels the polarity sensitivity of the disjunction word *huozhe* ‘or’, both children and adults are expected to generate a conjunctive entailment (the ‘neither’ interpretation). Therefore, both children and adults were expected to reject the target sentence (52) since the small pirate planted corals near the green mermaid. The fillers such as (53) were clearly true, so participants were expected to accept them. Taken together, the expected number of ‘Yes’ and ‘No’ responses was counterbalanced across trials.

#### Results and Discussion

**FIGURE 5:**
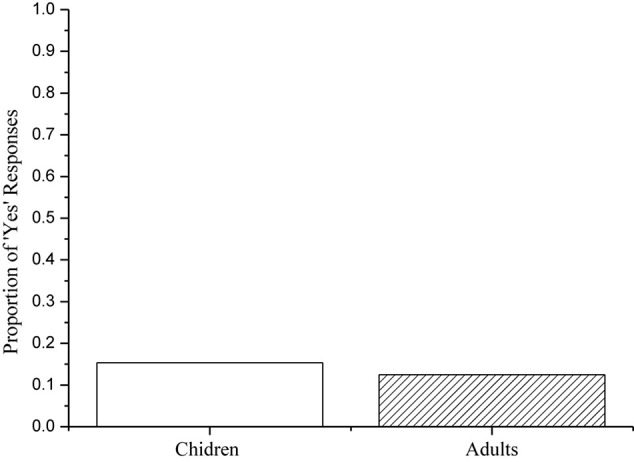
Children’s and adults’ percentages of ‘Yes’ responses to the target sentences.

Children accepted filler sentences like (53) 100% of the time. Adults accepted them 95% of the time. Therefore, the data of both groups were included in the analysis. A summary of children’s and adults’ responses to the targets is provided in Figure 5.

As shown in Figure 5, children rejected the target sentences 86% of the time, and adults rejected them 88% of the time. When asked for justifications for their rejections, both children and adults made reference to the fact that the other character also performed one of the two actions mentioned in the target sentences. For example, both children and adults justified their rejections to (52) by pointing out the fact that the small pirate also planted corals near the green mermaid. The findings indicate that the children and adults interpreted disjunction *in situ* in sentences with covert external negation, resulting in a ‘neither’ interpretation. A Mann-Whitney test on the No-responses to the target sentences revealed no significant difference between groups (*z* = 0.607, *p* = 0.544). Both Mandarin-speaking children and adults generate a conjunctive entailment (the ‘neither’ interpretation) in response to the target sentences. This finding confirmed our experimental hypothesis that external negation cancels the polarity sensitivity of the disjunction word *huozhe* ‘or.’

### Experiment 4

Experiment 4 investigated if both Mandarin-speaking children and adults negate but not cancel Free Choice Inferences in sentences with external negation. Compared with Experiment 3, Experiment 4 added the deontic modal verb *keyi* ‘is allowed to’ into the equation, as illustrated in (51), here repeated as (54).

(54) Zhiyou Zhangsan keyi chi yidalimian huozhe shousi.
Only Zhangsan may eat pasta or sushi
‘Only Zhangsan is allowed to eat pasta or sushi.’

In (54), the focus adverb *zhiyou* ‘only’ contributes two meaning components: a presupposition and an assertion. The presupposition expresses that Zhangsan is allowed to eat pasta or sushi. Due to the presence of the modal verb, the presupposition licenses a Free Choice Inference: *Zhangsan is allowed to eat pasta and is allowed to eat sushi*. The assertion entails that it’s not true that anyone other than Zhangsan is allowed to eat both pasta and sushi. In this sense, the focus adverb introduces covert external negation. If external negation cancels the polarity sensitivity, the disjunction word *huozhe* ‘or’ will be interpreted *in situ*. The result is a ‘not both’ interpretation. On this interpretation, the sentence is judged to be true even if someone other than Zhangsan is allowed to eat pasta, but not sushi, or sushi, but not pasta. Therefore, the experimental hypothesis was that both Mandarin-speaking children and adults would judge (54) to be true in circumstances that are consistent with the weaker ‘not both’ interpretation, rather than the stronger ‘neither’ interpretation that was expected in Experiment 3.

#### Participants

Twenty-five Mandarin-speaking children participated in the experiment, and they ranged in age from 4;1 to 5;2, with a mean age of 4;6. The child participants were recruited from a kindergarten affiliated with Beijing Language and Culture University, Beijing, China. We also tested 20 Mandarin-speaking adults, who were undergraduates at Hubei University of Technology, Wuhan, China.

#### Procedures

Participants were tested with the same methodology and procedures as Experiment 1.

#### Materials

We used the same test stories as Experiment 3. Participants heard a total of eight sentences: Four were targets and four were fillers. The eight sentences were evenly distributed across the same four test stories so that participants judged one target and one filler after each story. The target and filler items were presented in a pseudo-random order immediately after Mr. Owl proclaimed the rules. For example, on the typical trial, although Mr. Owl had proclaimed the rules, the two pirates were very forgetful. When they were about to start the game, they asked the puppet to remind them of the rules. At that point, the puppet presented the target in (55) and the filler in (56).

(55) Zhiyou dahaidao keyi zai hongse huozhe lüse meirenyu bianshang zhong shanhu.
Only big pirate may at red or green mermaid side plant coral
‘Only the big pirate is allowed to plant corals near the red or the green mermaid.’

(56) Zhiyou xiaohaidao keyi zai lüse meirenyu bianshang zhong shanhu.
Only small pirate may at green mermaid side plant coral
‘Only the small pirate is allowed to plant corals near the green mermaid.’

#### Predictions

We anticipated that participants would generate a negated Free Choice Inference (the ‘not both’ interpretation) in response to the target sentences such as (55). For example, participants were expected to assign a ‘not both’ interpretation to (56) if they negated the FCI. Therefore, they were expected to accepted (55) as the small pirate is allowed to plant corals near the green mermaid. The fillers such as (56) were clearly false, so participants were expected to reject them. Considered together, the expected ‘Yes’ and ‘No’ responses were counterbalanced across trials.

#### Results and Discussion

**FIGURE 6:**
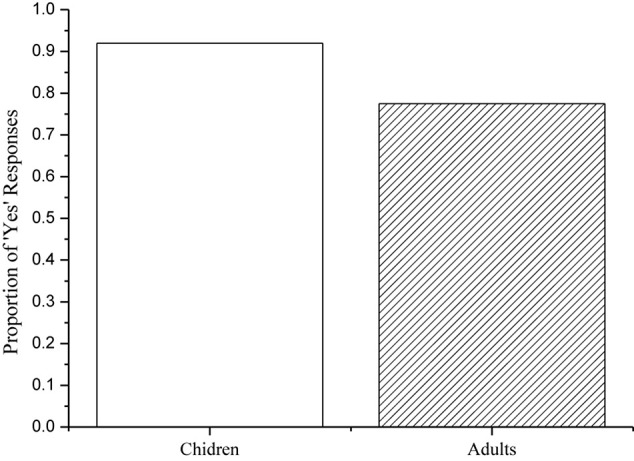
Children’s and adults’ percentages of ‘Yes’ responses to the target sentences.

Children correctly rejected fillers like (56) 97% of the time, and adults did so 100% of the time. Therefore, all their data were included in the final analysis. Figure 6 provides a summary of both children’s and adults’ responses to the target sentences.

As indicated in Figure 6, children accepted the target sentences 92% of the time, and adults accepted them 78% of the time. A Mann-Whitney test on the Yes-responses to the target sentences revealed no significant difference between groups (*z* = 1.474, *p* = 0.140). Notably, individual analysis showed that three of the 20 adults consistently rejected the target sentences, suggesting that they cancel Free Choice Inferences in sentences with external negation. In addition, two of the 20 adults rejected the target sentences 25% of the time, and another two did so 50% of the time. These adults’ behavior reveals that they more or less appear to cancel FCIs in sentences with external negation. Compared with children, adults had 14% reduction in the acceptance rate of the target sentences. This unexpected difference between children’s and adults’ behavior reveals that children are sometimes more logical than adults in the interpretation of logical expressions (see e.g., Noveck, 2001). Nonetheless, the majority of the child and adult participants assigned a ‘not both’ interpretation to the target sentences. Recall that both children and adults assigned a ‘neither’ interpretation to the target sentences in Experiment 3. The only difference between the two experiments is that the target sentences in Experiment 4 contained an additional modal verb. Considered together, the findings support the common assumption that modal verbs license FCIs (see, e.g., Fox, 2007; Chierchia, 2013). Furthermore, the findings confirmed our experimental hypothesis that FCIs are negated, but not canceled in sentences with external negation.

## General Discussion and Conclusion

The present study gave rise to four main findings. First, Mandarin-speaking children computed a conjunctive entailment (the ‘neither’ interpretation) in response to sentences with internal negation, a modal verb and disjunction, whereas adults assigned a ‘not both’ interpretation to the same sentences. Second, English-speaking adults distinguished the semantic differences between internal and external negation. Third, both Mandarin-speaking children and adults assigned a ‘neither’ interpretation to disjunctive sentences with external negation, introduced by the focus adverb *zhiyou* ‘only’. Fourth, when a modal verb was added into the equation, both Mandarin-speaking children and adults generated a negated FCI (the ‘not both’ interpretation).

Considered together, the findings reveal that 5-year-old Mandarin-speaking children already have the following linguistic knowledge in place. First, external negation cancels the polarity sensitivity of the Mandarin disjunction word *huozhe* ‘or’. Second, Free Choice Inferences are canceled in sentences with internal negation, but such inferences are negated, but not canceled in sentences with external negation. However, Mandarin-speaking adults add complexity to the picture as they do not cancel FCIs in sentences with internal negation. This is because Mandarin-speaking adults analyze disjunction as a PPI and consequently interpret disjunction as taking scope over internal negation and the modal verb. This results in a ‘not both’ interpretation’. To conclude, our findings support the distinction between internal and external negation (Karttunen and Peters, 1979; Ladusaw, 1980; Bochvar and Bergmann, 1981; Horn, 1985, 2001; Schwarz and Bhatt, 2006; Bar-Asher Siegal, 2015).

Two questions remain: First, why are Free Choice Inferences canceled in sentences with internal negation, but negated in sentences with external negation? To explain why, let’s consider the simple affirmative sentence with disjunction, as in (57).

(57) Rose ordered sushi or pasta.

Sentences like (57) invite an exclusivity inference. In classical logic, a formula with disjunction in the scope of negation, ¬ (*p* ∨ *q*), entails a conjunction of negative formulas, (¬ *p* ∧¬ *q*). Let us call this the conjunctive entailment of a negated disjunction. In English, negative sentences with disjunction generate a conjunctive entailment. For example, sentence (58) and (59) are judged to be true in the same circumstances.

(58) Rose did not order sushi or pasta.
(59) Rose did not order sushi and Rose did not order pasta.

In example (60), a modal verb has been added to the equation.

(60) Rose was allowed to order sushi or pasta. (She was free to choose).

Sentence (60) is no longer has that exclusivity inference associated with (57). Instead, (60) makes a conjunctive (Free Choice) inference. It is important not to confuse inferences and entailments. For one thing, inferences are defeasible; they can be negated without contradiction. The negation of an entailment, by contrast, results in a contradiction. Another distinctive feature of inferences is that they are canceled under internal negation. Sentence (61) is the negation of (60). The interpretation assigned to (61) is a conjunctive entailment (62), not the negation of the conjunctive inference, which is paraphrased in (63).

(61) Rose was not allowed to order sushi or pasta.
(62) Rose was not allowed to order sushi and Rose was not allowed to order pasta.
(63) Rose was not allowed to order both sushi and pasta.

Here is one place that the distinction between internal and external negation becomes relevant. With external negation, as (64), the conjunctive (Free Choice) inference licensed by (60) is negated. Sentence (64) is judged to be true in a context where Rose is allowed to order pasta, but not sushi. Putting it the other way around, (64) does not generate a conjunctive entailment. In sum, that’s why FCIs are cancelled in sentences with internal negation, but such inferences are cancelled with external negation.

(64) It did not turn out that Rose was allowed to order sushi or pasta. (She was only allowed to order sushi).

Secondly, how do children acquire the different patterns of entailments and inferences that we observed in the present study? A nativist account to language acquisition contends that children are endowed with the linguistic knowledge of the meanings of basic logical expressions and these meanings are, for the most part, consistent with the truth conditions associated with the corresponding expressions in classical logic (Crain, 2008, 2012; Crain and Khlentzos, 2008, 2012). The nativist account predicts that children draw upon the semantic meanings of logical expressions that are considered to be part of a Universal Grammar (Chomsky, 1965) in the course of language development (Crain and Pietroski, 2001; Crain et al., 2005).

By contrast, a usage-based approach to language acquisition maintains that children acquire linguistic knowledge from experience, using general cognitive mechanisms (e.g., Cameron-Faulkner et al., 2007; Lieven and Tomasello, 2008). In other words, the usage-based account indicates that there is a strong correspondence between the linguistic forms in the input and the child’s emergent linguistic knowledge. Therefore, from a usage-based perspective, children are predicted to acquire the different patterns of inferences and entailments that we have observed by attending to the corresponding statistical regularities in the input.

To assess these two predictions, let’s revisit the interpretations assigned by both children and adults in response to sentences with internal negation, in which negation appeared in the same clause as the deontic modal verb *beiyunxu* ‘was allowed to’ and the disjunction word *huozhe* ‘or’. The child participants computed a conjunctive entailment (the ‘neither’ interpretation). By contrast, the adult participants generated a ‘not both’ interpretation. Therefore, it seems very unlikely that children’s computation of conjunctive entailments as such would be drawn from adult input, given the fact that children assign a different interpretation from adults to the target sentences.

Consider the interpretations assigned by both children and adults in response to sentences with external negation. In the first kind of test sentences with external negation, the focus adverb *zhiyou* ‘only’ was combined with the disjunction word *huozhe* ‘or’. In response to such sentences, both children and adults generated a conjunctive entailment (the ‘neither’ interpretation). In the second kind of test sentences with external negation, the focus adverb *zhiyou* ‘only’ was combined with the deontic modal verb *keyi* ‘is allowed to’ and the disjunction word *huozhe* ‘or’. In response to such sentences, both children and adults computed a negated FCI (the ‘not both’ interpretation). The usage-based account may argue that children acquire the conjunctive entailment and the negated FCI via adult input since they share the same interpretations as adults. If there were sufficient quantities of adult utterances containing the combination of *zhiyou + huozhe* or *zhiyou + keyi + huozhe*, it would be likely for children to learn the interpretations from adult input. To test this possibility, we did a corpus analysis. We searched the seven Mandarin corpora on the Child Language Data Exchange System (CHILDES) database and the BJCELA corpus. There were only 675 tokens of parental utterances with *keyi*, 21 tokens of parental utterances with *huozhe* and 54 tokens of parental utterances with *zhiyou.* However, no utterances contained the combination of *zhiyou + huozhe* or *zhiyou + keyi + huozhe* in these corpora^10^. Therefore, the paucity of the relevant adult input reveals that it is unlikely that children learn either the conjunctive entailment or the negated FCI merely via adult input.

Taken together, the corpus analysis indicates that it is implausible to postulate that children acquire the different patterns of entailments and inferences merely from primary linguistic experience. Rather, the findings appear to support the nativist account to the acquisition of logical expressions. In particular, children’s non-adult interpretation of sentences with disjunction, internal negation and a modal expression reflects an initial setting of a lexical parameter. According to the parameter, the Mandarin disjunction word *huozhe* ‘or’ is a PPI for adults, but not for young children (e.g., Crain et al., 2016). That is, the lexical parameter of Mandarin disjunction has two values. Adults take the OR = +PPI value, on which the disjunction word *huozhe* is interpreted as taking scope over internal negation and the modal verb, resulting in a ‘not both’ interpretation. By contrast, children initially take the OR = −PPI value, on which the disjunction word *huozhe* is interpreted *in situ*, resulting in a ‘neither’ interpretation. Presumably, children acquiring Mandarin are able to access both interpretations of sentences that contain disjunction, internal negation and a modal expression. To avoid learnability problems, however, the language acquisition device (LAD) enforces an initial preference for the strongest interpretation of such sentences. According to the Semantic Subset Principle, children are compelled to adopt a subset value of certain parameter (e.g., the Mandarin disjunction parameter), which makes a sentence true in a subset of the circumstances that make it true on the alternative value (Crain and Thornton, 1998; Crain, 2012). Therefore, children acquiring Mandarin are expected to initially assign a ‘neither’ interpretation to sentences containing disjunction, internal negation and a modal expression. Assuming that the preferred scope assignment between disjunction and internal negation takes time to reverse, children will be compelled by positive evidence to converge on the ‘not both’ interpretation adopted by adults. In addition, children are expected to rely on the syntactic process of MERGE to combine meanings from sentences with external negation compositionally. Mechanisms like MERGE and compositionality enable children to derive the negated FCIs from sentences with external negation, as in the experimental test sentences of the present study (Chomsky, 1995).

## Data Availability Statement

The raw data supporting the conclusions of this article will be made available by the authors, without undue reservation.

## Ethics Statement

The studies involving human participants were reviewed and approved by Human Research Ethics Sub-committe, Faculty of Human Science, Macquarie University, Sydney, Australia. Written informed consent to participate in this study was provided by the participants’ legal guardian/next of kin.

## Author Contributions

HQH ran all of the experiments and wrote the first draft of the manuscript. PZ and SC edited the manuscript. All authors contributed to the article and approved the submitted version.

## Conflict of Interest

The authors declare that the research was conducted in the absence of any commercial or financial relationships that could be construed as a potential conflict of interest.

## Footnotes

1 See, e.g., Collins and Postal (2014) *Classical NEG Raising*. The MIT Press.

2 *Mei* and *bu* are both primary negation markers in Mandarin Chinese. Roughly, *bu* is used to negate habitual/future/volitional situations, whereas *mei* is used to negate the completion of an event (e.g., Li and Thompson, 1980).

3 Note that *bu-shi* in Mandarin Chinese is generally assumed to be an external negation marker (e.g., Ernst, 1995; Hsieh, 1996, 2001; Lee and Pan, 2001; Lin, 2003; Huang, 1988a,b).

4 As an exclusion criterion, participants who scored less than 80% correct on the filler or control sentences would be excluded in the final data analysis. The exclusion criterion was applied in all of the four experiments in the present study.

5 For convenience of comparing the data across experiments, we consistently graphed the proportion of ‘Yes’ responses in all of the four experiments in the present study.

6 It is worth noting that we have the same scope assignment of the three operators (∼, ♢, ∨) in sentences like (46) and (48), namely NOT >ALLOW >OR, but a different interpretation when it comes to FCIs. Specifically, negation in sentences like (48) is interpreted as taking a wide scope over the clause that contains the modal verb and disjunction. The clause gives rise to a FCI in its local domain. As a result, the FCI is negated, rather than cancelled, resulting in a ‘not both’ interpretation. By contrast, negation in sentences like (46) is interpreted as taking an immediate scope over the modal verb and disjunction, so the FCI is cancelled, resulting in a ‘neither’ interpretation. Notably, it would be interesting to see whether young children have the same interpretations as adults when processing the above-mentioned sentences that contain more than one scope-bearing element.

7 Note that although *bu-shi* is commonly assumed to be an external negator in Mandarin Chinese (see e.g., Ernst, 1995; Hsieh, 1996, 2001; Lee and Pan, 2001; Lin, 2003; Huang, 1988a,b), it has been documented that Mandarin-speaking children are able to understand covert external negation, introduced by the focus adverb *zhiyou* ‘only’ (e.g., see Zhou and Crain, 2010). Therefore, it would be more interesting to look at child grammar of external negation hidden in a linguistic structure with the focus adverb *zhiyou*. That’s why the choice of form of external negation is different across Experiments 2 versus 3/4.

8 It should be noted that *zhiyou* and *zhi* are two variants of the focus operator *only* in Mandarin Chinese. *Zhiyou* is often associated with the focused element in the pre-subject position or the predicate position. For instance, Mandarin speakers can say *Zhiyou Zhangsan chi-le shousi* ‘Only Zhangsan ate sushi’ and *Zhangsan zhi(you) chi-le shousi* ‘John only ate sushi’. When the focus operator appears in the preverb position, Mandarin speakers tend to omit the second morpheme *you*, for example, *Zhangsan zhi chi-le shousi ‘*Zhangsan only ate sushi’. Nevertheless, there is no interpretative differences between *zhi* and *zhiyou* when both appears in the preverb position.

9 Note that the puppet’s ignorance of the results of the coral-planting made the use of disjunction felicitous.

10 It should be noted that the target linguistic structures might occur in larger corpora, but the paucity of such linguistic structures in the relatively small corpora that we searched indicates that they are unlikely to have high frequency even in larger corpora.
